# Quantum theories of consciousness: a critical review of feasibility, philosophical sufficiency, and empirical testability

**DOI:** 10.3389/fpsyg.2026.1730965

**Published:** 2026-04-29

**Authors:** Xun Ma, Aoping Wang

**Affiliations:** Department of Philosophy, Xiamen University, Xiamen, China

**Keywords:** bridge principle, consciousness, explanatory gap, hard problem, quantum

## Abstract

Explaining subjective experience (the “hard problem”) remains a central challenge in consciousness science. Research on “quantum consciousness” has grown rapidly in recent years, yet a substantial subset merely links terms such as collapse, superposition, and entanglement to qualia in verbal maneuvers that lack testable mechanistic commitments. This article adopts a theory-first critical-review framework and evaluates three classes of quantum theories of consciousness along three axes: (1) physical feasibility in warm, wet neural tissue strongly coupled to its environment; (2) philosophical sufficiency, i.e., a bridge from posited quantum processes to phenomenal character; and (3) empirical testability against classical alternatives. Based on the synthesized evidence, the present review suggests that further progress should prioritize experimentally tractable programs, in order to avoid the use of quantum terminology without mechanistic grounding in consciousness research and to convert debates into discriminating empirical programs.

## 1 Introduction and problem diagnosis

Research on consciousness is commonly divided into the ‘easy problems’ and the ‘hard problem’ (Chalmers, 1995), a distinction that is often associated with Block’s account of access consciousness and phenomenal consciousness (Block, 1995). The easy problems primarily concern access-conscious capacities, namely the availability of information for report, reasoning, and the control of action. By contrast, the hard problem concerns phenomenal consciousness and the what-it-is-like aspect of experience (Nagel, 1974), including its qualitative character, such as the redness of red or the painfulness of pain. Many naturalistic theories of consciousness face a bottleneck at the hard problem, namely an “explanatory gap” between physical/functional descriptions and phenomenal subjectivity (Levine, 1983). Against this backdrop, some researchers have turned to the “non-classical” features of quantum mechanics in the hope of breaking the impasse, giving rise to so-called quantum approaches to consciousness (Atmanspacher, 2024; Hameroff and Penrose, 1996; Penrose, 2016; Stapp, 1982). They suppose that mechanisms such as ‘quantum superposition’, ‘entanglement’, or ‘wavefunction collapse’ may participate in conscious processing and thereby offer new solutions to the hard problem of consciousness. However, we note that a considerable portion of recent discussions labeled “quantum consciousness” are terminology-driven rather than mechanism- or evidence-driven: quantum-theoretical terms are often invoked in a largely narrative or analogical manner without specifying their precise physical meaning or empirical applicability, and an “account” of consciousness is then proclaimed (Atmanspacher, 2024; Tegmark, 2000). This practice often lacks rigorous argumentation, remains insufficiently constrained by clear mechanisms or empirical support, and therefore does not yet provide a substantive solution to the problem of consciousness.

We hold that any reasonable quantum model of consciousness (indeed, any reasonable theory of consciousness) should at least satisfy three basic commitments. (i) Physical feasibility: the posited quantum processes must be realizable in warm, wet neural tissue that is strongly coupled to its environment (i.e., an open, decohering regime), and embedded within densely and recurrently interconnected neuronal networks (Tegmark, 2000; Koch and Hepp, 2006; Schlosshauer, 2007; Averbeck et al., 2006). (ii) Philosophical sufficiency: there must be a bridge principle from the posited quantum processes to the characteristics of subjective phenomenology. It is not enough to assert that the existence of some quantum phenomenon/process entails the existence of experience, for then the hard problem remains (Chalmers, 1995, 1997).^1^ (iii) Empirical testability: the theory should yield predictions that discriminate it from classical alternative mechanisms and from rival theories of consciousness, and these predictions should be empirically testable and replicable (Doerig et al., 2021).

Quantum-related phenomena in biological systems are, of course, not rare. For example, quantum-coherent energy transfer in photosynthesis (Engel et al., 2007; Cao et al., 2020; Dudhe et al., 2022) and spin-chemical mechanisms in avian magneto reception (Hore and Mouritsen, 2016; Xu et al., 2021) have been reported. While these results establish that quantum effects can play functional roles in biological efficiency, they do not by themselves entail that quantum processes are constitutive of consciousness. Quantum consciousness proposals make the stronger claim that specific quantum degrees of freedom in the brain—that is, particular quantum-state variables or dimensions of physical variation, such as nuclear spins or electronic/dipolar states—are not merely enabling conditions, but help determine or realize subjective experience itself (Hameroff and Penrose, 2014; Fisher, 2015). The present review focuses on this stronger, constitutive sense. Accordingly, whether any proposed quantum phenomenon is methodologically relevant to mind-related brain activity must be assessed against these three commitments.

On this basis, the present review evaluates several quantum theories or mechanisms of consciousness that have attracted sustained attention in the literature because they appear relatively promising in meeting these three principles. We critically assess three programs that have generated sustained empirical or methodological debate and that specify clear neural/biological substrates with repeatable experimental pathways. In what follows, we use the term “Family” to denote a cluster of related programs that share a core mechanistic hypothesis and experimental pathway, rather than a single, fully unified theory. *Family A* covers the Penrose-Hameroff Orch OR (Orchestrated Objective Reduction) framework together with its microtubule-based quantum-optical extensions; (Hameroff and Penrose, 1996; Hameroff and Penrose, 2014). *Family B* comprises Fisher’s nuclear-spin/Posner-molecule hypothesis and subsequent developments in the same line (Fisher, 2015).; *Family C* groups Kerskens’ reports of macroscale “non-classical” brain signals in quantum-optical and MRI (magnetic resonance imaging) paradigms, along with closely related follow-up work (Kerskens and López-Pérez, 2022; López Pérez et al., 2023). Because the literature surrounding microtubule-based models (Family A) is currently more extensive and experimentally developed than that of the other two approaches, this family receives correspondingly more extensive coverage in the present review.

This is a selective, theory-driven review rather than a systematic review. The scope of this review is deliberately restricted to quantum proposals that posit specific quantum degrees of freedom in identifiable neural or biological substrates and offer at least some operational experimental pathway for testing; this is why the discussion focuses on the three theory families examined below. We therefore do not survey the broader landscape of quantum biology (e.g., photosynthesis, magnetoreception) except as background, nor do we cover more metaphorical or purely interpretive “quantum mind” speculations that lack a worked-out biophysical implementation in the brain, or general panpsychist/cosmopsychist views where quantum theory plays only an abstract or analogical role. These exclusions are methodological rather than evaluative: the three-axis framework developed here is tailored to mechanism-driven, testable models, and other approaches would require a different set of questions and criteria^2^.

## 2 Family a: microtubule/Orch OR models and quantum-optical extensions

Theories in Family A are represented by the Orch OR (Orchestrated Objective Reduction) model, which holds that consciousness arises from quantum coherence and gravitational objective-collapse events in neuronal microtubules (Hameroff and Penrose, 2014). On this view, microtubules serve as a substrate for quantum computation. Individual tubulins, or helical topological pathways of many entangled tubulins along microtubule lattice geometry (the latter potentially resistant to quantum errors), can function as qubits. Coordinated quantum collapses within these microtubule networks then give rise to ordered patterns of neural activity and conscious experience. If the model is correct, general anesthesia would be expected to induce unconsciousness by acting on microtubular quantum processes; for example, volatile anesthetics should attenuate or suppress certain quantum vibrational or optical signals in microtubules (Craddock et al., 2014). In recent extensions, it has been proposed more concretely that such putative quantum effects can be detected by measuring spontaneous fluorescence or vibrational signals of tryptophan residues within microtubules (Kalra et al., 2023).

### 2.1 Thesis and predictions

The core claim of Orch OR is that consciousness is not produced solely by classical neural-network activity but involves quantum-coherent processes in the subcellular structure of microtubules. When a sufficient number of tubulin subunits within a microtubule or group of entangled microtubules reach a threshold of quantum superposition at times t = ℏ/E_G_, the overall wavefunction undergoes objective reduction. In the formula, ℏ is the Planck-Dirac constant and E_G_ the gravitational self-energy of separating superpositioned mass, or its equivalent spacetime curvature, from itself. In Orch OR, E_G_ is calculated for the number of entangled tubulins in superposition, and the corresponding time (t) is then derived for OR (“quantum collapse”) and the occurrence of a moment of conscious experience. Initially Hameroff and Penrose (1996) set t at 25 milliseconds (msec) for 40 Hz gamma synchrony EEG (electroencephalography), the best correlate of consciousness. For t = 25 msec, E_G_ turned out to be 2 × 10^10^ tubulins, a few thousand neurons’ worth, a rather small percentage of total brain neurons (~10^11^) and tubulins (~10^19^). Moreover 25 msec for 40 Hz is a long time to avoid decoherence.

Beginning in 2013 Bandyopadhyay’s group (Sahu et al., 2013a, 2013b, 2014, Saxena et al., 2020; Ghosh et al., 2022) reported self-similar coherent resonance oscillation patterns (triplets-of-triplets) in microtubules in kilohertz, megahertz, gigahertz and terahertz frequencies, microtubules apparently functioning as fractal time crystals (Hameroff and Lauretta., 2026). In 2014, Orch OR theory (Hameroff and Penrose, 2014) began to use the coherent microtubule frequencies reported by Bandyopadhyay’s group to set the relevant time parameter t for Orch OR events (i.e., the frequency of putative quantum collapses). For t = 10^−7^ s(sec), E_G_ corresponds to 10^15^ tubulins—about one ten-thousandth of the total number of tubulins in the brain. On this timescale, Orch OR events would occur at 10 megahertz, implying that coherence would need to be maintained for only 10^−7^ s, which is argued to be comparatively feasible. But cognitive events like visual scenes, evoked potentials and EEG take several 100 msec, 5 orders of frequency slower. Hameroff and Penrose (2014) suggested negative resonance, or interference beats among slightly different microtubule oscillations (e.g., in mixed polarity anti-parallel microtubules found only in neuronal dendrites and soma) would accommodate interference and transcend scale, ‘downshifting’ to EEG frequencies.

In addition, the theory emphasizes that the selective suppressive effect of anesthetics on consciousness can be explained by quantum mechanisms: volatile anesthetic molecules bind in hydrophobic pockets of tubulin, disrupting *π*-electron resonances or vibrational modes and thereby degrading microtubular quantum coherence to produce loss of consciousness (Craddock et al., 2014). Accordingly, if microtubular quantum processes are necessary for consciousness, then (a) different anesthetics should interfere with quantum vibrational or excited-state signals in microtubules in a dose-dependent manner, and proportional to potency of each anesthetic in immobilizing animals and humans, the consistent with the Meyer–Overton correlation^3^; (b) conversely, drugs that stabilize microtubules should delay or mitigate anesthetic effects; and (c) one might detect marked differences in microtubule-related optical or vibrational indices between waking and anesthetized brains.

### 2.2 Axis I: physical feasibility

A primary challenge for Family A is whether microtubules can sustain sufficiently stable quantum coherence to support Orch OR-style computation. Given typical microtubule dimensions (≈25 nanometer (nm) diameter) and conditions (≈37 °C with complex ionic/molecular interactions), quantum states are expected to decohere on extremely short timescales (Tegmark, 2000). Based on thermal fluctuations and environmental scattering, Tegmark (2000) estimated that quantum coherence times for electrons or protons in microtubules are only on the order of 10^−13^ s. But Tegmark used a superposition separation distance of 10^−18^ meters instead of one femtometer 10^−15^ meters, stipulated in Orch OR. Hagan et al. (2002) used Tegmark’s same formula corrected for separation distance and calculated 10^−16^ s decoherence time, or coherence limit. Experimentally Kakati et al. (2024) showed a microtubule quantum state persisting for $10^{-8}$ sec, and longer quantum optical states have been reported. Thus 10^−6^ to 10^−8^ s is a reasonable range for microtubule decoherence (coherence limit), and briefer coherence times (10^−9^ to 10^−12^ s) are also feasible for Orch OR in frequencies from gigahertz to terahertz. Bandyopadhyay’s coherent oscillations may be quantum above a critical frequency, and in the Orch OR theory objective reduction occurs at times t = ℏ/E_G_ and t and E_G_ may vary (inversely to each other), and Orch OR events at different frequencies are suggested in Orch OR to resonate, and interfere somewhat like notes and chords in music (Hameroff and Penrose, 2014; Sahu et al., 2013a; Saxena et al., 2020).

In recent years, experimental biophysics has supplied *in vitro* clues supportive of coherence feasibility. For instance, using microtubules’ intrinsic tryptophan fluorescence lifetimes, Kalra et al. (2023) found that photo-excited electronic energy can migrate over ≈6.6 nm persisting $10^{-8}$ sec along the microtubule lattice. This pattern is difficult to capture in terms of purely classical Förster-type incoherent hopping alone, and is therefore more naturally interpreted as involving quantum-coherent exciton migration along the microtubule lattice (Kalra et al., 2023; Kakati et al., 2024). Adding anesthetics (isoflurane or etomidate) significantly shortens this excitonic diffusion length and coherence time (Kalra et al., 2023; see also Kakati et al., 2024). In addition, Babcock et al. (2024) report ultraviolet superradiance in large microtubule networks: tens of thousands of tryptophan residues exhibit cooperative radiative dynamics with strongly superradiant states, leading to an enhanced fluorescence quantum yield in microtubule assemblies. These findings indicate that microtubules and related intracellular structures exhibit certain quantum-optical properties. However, it should be noted that most of the existing evidence still derives from in vitro or ex vivo preparations, and its applicability to the intact *in vivo* brain remains uncertain. Notably, recent work by Bandyopadhyay and colleagues on cultured living neurons has shown, via causal manipulation of microtubule resonance frequencies, that microtubule oscillations can modulate spiking activity at the neuronal membrane and give rise to correlated patterns spanning multiple neurons (Saxena et al., 2020; Singh et al., 2021a, 2021b; Ghosh et al., 2022). These findings provide important support for the functional relevance of microtubule-associated resonances under physiological conditions, yet the stability, reproducibility, and conformity of such phenomena to the specific quantum conditions required by Orch OR in the intact brain remain to be independently established. Moreover, for Orch OR’s objective reduction to occur, the mass scale and duration of coherent superposition must satisfy Penrose’s OR condition, something that remains highly uncertain in the neuronal milieu (Tegmark, 2000; Schlosshauer, 2007).

### 2.3 Axis II: philosophical sufficiency

Even granting ongoing quantum processes in microtubules, Family A faces a bridging challenge in philosophical explanation: how do physical processes at the quantum level give rise to the nature of subjective experience? Current expositions of Orch OR tend to remain at the level of an intuition: if there are quantum processes, novel conscious states may arise, without stating a clear rule of derivation from quantum-state dynamics to the what-it-is-likeness of experience. For example, which microtubular quantum collapses would correspond to the particular qualitative texture of a given quale (e.g., the redness of red)? Why would a 40-Hz collapse rhythm yield a unified, fluent stream of consciousness rather than a flicker? Empirical work on discrete perceptual windows and proposals that *γ*-frequency Orch OR events might serve as candidate integration cycles (VanRullen and Koch, 2003; VanRullen, 2016; Wiest and Puniani, 2025) arguably mitigate the intuitive “flicker” objection by suggesting a structural fit at the level of temporal organization.^4^ Quantum processes, as such, are physical events. They do not entail subjectivity on their own. Critics therefore note that even if Orch OR were to demonstrate distinctive microtubular physics, this might at most identify a mechanism associated with consciousness, not a solution to why first-person experience has its particular character (Levine, 1983). Recent work has suggested that microtubules may exhibit “fractal time crystal” behavior, that is, temporally ordered and self-similar oscillatory dynamics across scales (Hameroff and Lauretta., 2026). Such a proposal, if independently confirmed, could strengthen the case for distinctive microtubular dynamics at the physical level; however, it would still not by itself supply a bridge principle connecting those dynamics to determinate phenomenal character.

Proponents of Orch OR nevertheless offer a set of familiar lines of connection between the theory and the “hard problem” of consciousness. First, Gödel’s theorem (Gödel, 1931) indicates consciousness is non-computable, i.e., non-algorithmic, allowing for some outside system to judge or validate correctness of a mathematical theorem (Penrose, 2016). Penrose applied this to conscious understanding, and sought a system outside classical physics in quantum collapse of the wavefunction. Second, invoking Alfred North Whitehead’s notion of ‘occasions of experience’ (Whitehead, 1929), that is, elementary events of experience in Whitehead’s process philosophy, Penrose first characterized superposition as separated curvatures in spacetime geometry, with collapse occurring at an objective threshold related to the quantum uncertainty principle. At that instant a conscious (or proto-conscious) moment of experience would occur, i.e., the accessing of qualia embedded in spacetime geometry, and collapse moments are taken to be similar to Whiteheadian ‘occasions of experience’in a wider field of protoconscious experience. Third, regarding the “conscious observer”: some authors claim that conscious observation causes collapse (e.g., Von Neumann, 2018; Wigner, 1961). The double-slit experiment is often invoked in support of this interpretation, since interference disappears when which-path information is obtained (Schlosshauer, 2007). However, this move effectively places consciousness outside ordinary physical explanation and, by itself, does not explain why superposed states should give rise to experience or how consciousness is related to superposition in the first place (Schlosshauer, 2007; Atmanspacher, 2024). Penrose turned this around: collapse occurred spontaneously and caused, or was equivalent to, consciousness. Orch OR accommodates this in the following way: a conscious observer having repetitive superpositions and Orch OR events observes the superposition, and at some point during each Orch OR event in the observer’s brain microtubules, the E_G_ of the observer would entangle with the observed superposition to reach threshold, causing collapse and conscious observation of a particle. Fourth, the theory appeals to entanglement: if the observer becomes entangled with the observed superposition, Orch OR proponents suggest that the relevant phenomenal contents (qualia) may be, in an intended sense, “shared” across the entangled system. Fifth, more generally, many panpsychist/cosmopsychist frameworks treat consciousness as fundamental; Orch OR aligns with this orientation by locating proto-phenomenal properties in fundamental spacetime geometry and treating OR as the access mechanism. A recent proposal by Wiest and Puniani (2025) is an important step here: interpreting Orch OR within a panprotopsychist framework, he advances a specific psychophysical bridging principle according to which the entanglement structure of an objectively unified Orch OR state naturally corresponds to unified conscious contents, thereby addressing the binding problem and offering a non-epiphenomenalist account of the evolution of consciousness. While this framework arguably enhances the philosophical sufficiency of Orch OR with respect to unity and causal efficacy, it still relies on substantial metaphysical postulates and leaves questions about fine-grained qualitative character, phenomenal affect, and intentionality largely open. In this sense, the search for an explicit and broadly compelling bridge principle within Family A remains incomplete.

### 2.4 Axis III: empirical testability

A major strength of Family A is the progressive articulation of operational empirical criteria. One can, for example, test Orch OR via anesthetic modulation of microtubular quantum signals. Concretely, researchers can pre-register experiments using Förster resonance energy transfer(FRET)or time-resolved fluorescence to measure excitonic energy transfer or vibrational modes in microtubule networks, and compare how multiple anesthetics at varying concentrations affect these signal indices. If Orch OR holds, anesthetics should significantly and dose-dependently attenuate microtubular quantum signals rather than merely suppress nonspecific noise. Dose dependence should follow the Meyer-Overton correlation between anesthetic effects dampening quantum oscillations in microtubules, and clinical effects rendering animals and humans unconscious and unresponsive. The only such correlation thus far shown is for computer modeling of anesthetic inhibition of photon emission from tubulin (Craddock et al., 2017). In addition, treating model organisms with microtubule stabilizers (e.g., epothilone B) is predicted to delay the onset of anesthesia (Khan et al., 2024). This prediction has received initial support in rats: after administration of a microtubule stabilizer, animals show reduced sensitivity to isoflurane-induced loss of righting reflex (LORR), that is, they are harder to anesthetize (Khan et al., 2024). Li et al. (2025) broadly replicated and extended these effects with different microtubule-modulating drugs in mice, and Linganna et al. (2015) reported that prior exposure to taxane chemotherapy is associated with altered sensitivity to inhalational anesthetics in surgical patients. Taken together, these findings suggest that general anesthetics may, at least in part, act via microtubule-related mechanisms and strengthen the case for microtubules as functionally relevant anesthetic targets.

Nonetheless, further preregistered, sufficiently powered studies across independent laboratories, with transparent reporting of both positive and null results, are required to establish the robustness, specificity, and clinical relevance of these microtubule-based effects. Equally important is to record microtubule-linked signals alongside traditional neurobehavioral indices (e.g., EEG or arousal assessments) to establish associations between microtubular quantum markers and conscious state. If microtubular fluorescence lifetimes or vibrational modes observed in wakefulness diminish or vanish under anesthetic unconsciousness, and if such changes exceed classical thermal-noise explanations, this would constitute strong evidence for Orch OR (Kalra et al., 2023). Conversely, rigorous tests that fail to detect the predicted differences would demarcate the theory’s limits. In sum, Family A has proposed actionable experimental programs and already yielded some initially reproducible findings (e.g., fluorescence lifetimes), giving it an advantage in empirical testability over quantum consciousness proposals that remain purely at the level of hypothesis.

## 3 Family B: nuclear spins/Posner molecule hypothesis

Family B, proposed by physicist Fisher and colleagues, holds that quantum processes relevant to consciousness may reside at the level of nuclear spins rather than electrons or photons (Fisher, 2015; Ettenberg et al., 2020). The hypothesis focuses on the atomic nuclei of phosphorus-31 (^31^P), whose nuclear spin (the intrinsic quantum angular momentum of atomic nuclei) is 1/2 and which are mainly found in inorganic phosphate (Pi). Fisher’s proposal is that phosphate species can form intracellular clusters known as Posner molecule, a calcium–phosphate cluster structure originally described by Posner and Betts (1975). These clusters have the stoichiometry Ca_9_(PO_4_)_6_, where Ca denotes calcium and PO_4_ the phosphate group, and are complexed with calcium ions (Ca^2+^). In principle, such nanometer-scale clusters could sequester several nuclear spins in a protected environment. Owing to the symmetry and rigidity of the Posner structure, Fisher predicts that the enclosed nuclear spins may exhibit long-lived coherence (sec, minutes, or longer), potentially supplying a substrate for quantum information processing across neurons (Fisher, 2015). On this basis, Family B advances several testable predictions: (a) if nuclear spins contribute to neural function, isotopes (i.e., variants of the same chemical element differing in nuclear composition) with different nuclear spins should yield detectable physiological/behavioral differences. (b) In particular, lithium isotopes with distinct nuclear spins (e.g., ^6^Li^+^ vs. ^7^Li^+^) may differ in treating bipolar disorder or modulating neural activity, since lithium can influence downstream phosphorus metabolism (Ettenberg et al., 2020). (c) If two Posner molecules become entangled and then bind, they may simultaneously release Ca^2+^,triggering synchronized neurotransmitter release in a pair of spatially separated neurons; this is offered as a potential neural quantum correlation mechanism (Fisher, 2015).

### 3.1 Thesis and predictions

Fisher’s nuclear-spin hypothesis centers on the claim that biological phosphate processes, traditionally regarded as metabolic, may hide channels for quantum superposition and entanglement.

Concretely, in the cytosol, two phosphorus-containing molecules (e.g., pyrophosphate), under enzymatic action, complex calcium ions to form stable Posner clusters, each containing six ^31^P nuclei in inorganic phosphate (Pi). The nuclear spins of these ^31^P nuclei may, via chemical reactions, become entangled into three pairs, after which the Posner molecules diffuse and may even enter presynaptic terminals. If two entangled Posner molecules are taken up by different neurons, their entangled state collapses upon molecular binding, simultaneously releasing Ca^2+^, thereby causing synchronized neurotransmitter release in a pair of spatially separated neurons (Fisher, 2015). In principle, this mechanism could address the integration/binding problem, since entanglement provides a nonlocal linkage. Additional physiological-level predictions include: a spinful ^43^Ca isotope (nuclear spin 3/2) may differ from the common ^40^Ca (spinless) in its influence on conscious state and raising the calcium ion concentration in cerebrospinal fluid could enhance wakefulness. In sum, Family B sketches a pathway from quantum spins to neural population phenomena, and explicitly asserts that nuclear-spin quantum effects can be detected by altering elemental isotopes.

### 3.2 Axis I: physical feasibility

For Family B, the primary physical challenge is the actual existence and lifetime of Posner molecules and of nuclear-spin entanglement within them. Current chemical and biophysical studies diverge on the structure and dynamics of Posner molecules. Some calculations indicate that, under ideal conditions, the Ca_9_(PO_4_)_6_ cluster can form and provide good decoherence shielding for the six Pi nuclei (Swift et al., 2018). Subsequent work, however, suggests that under realistic conditions Posner molecules may favor lower-symmetry configurations, or even alternative calcium-phosphate cluster forms, such that intracluster nuclear-spin couplings are not perfectly screened and long-lived entanglement becomes harder to sustain (Player and Hore, 2018; Agarwal et al., 2023; Adams et al., 2025). For example, Player and Hore (2018), taking intracluster magnetic dipolar interactions into account, infer that the decoherence time of Pi-spin entanglement may be far shorter than earlier estimates—insufficient to reach the second-scale regime. On the experimental side, key assumptions also remain unverified: Chen et al. (2020) injected a calcium chelator and different calcium isotopes into the mouse lateral ventricle to test whether the depth of anesthesia would be altered. They found that lowering Ca^2+^ deepened anesthesia, whereas injecting ^43^Ca and ^40^Ca both reversed this effect, with no significant difference between the two isotopes (Chen et al., 2020). This last point matters: if nuclear-spin entanglement plays a role, ^43^Ca (with a nuclear magnetic moment) should differ from ^40^Ca, so the non-differentiation undermines the hypothesis. Moreover, although lithium isotope differences have been reported in animal behavior (Ettenberg et al., 2020), there is no direct evidence tying them to Posner nuclear spins: lithium may affect neural function via multiple classical routes.^5^ *In vitro* chemistry has, in a recent study, shown subtle lithium-isotope effects on calcium phosphate precipitation, interpreted by the authors as zero-point energy differences (Deline et al., 2023). Similarly, studies report that xenon isotopes with different nuclear spins (e.g.,^129^Xe vs. ^132^Xe) have different anesthetic potencies, not explained by classical polarizability (Li et al., 2018). While such findings support a role for nuclear spins in biology, the specific mechanisms may involve radical-pair-type quantum chemistry (Zadeh-Haghighi and Simon, 2023) rather than entanglement of Posner molecules. Essentially, the physical feasibility of Family B hinges on future direct observations or inferences of Posner existence and Pi coherence lifetimes.^6^ The current evidence is mixed: the idea is theoretically possible, but key parameters (structural symmetry, entanglement preservation) are contested, and there is currently no direct experimental observation of entanglement between nuclear spins in identified Posner molecules, either *in vitro* or *in vivo*.

Taken together, isotope findings at present admit several competing interpretations. (i) In a Posner-based picture, lithium or xenon isotope effects would arise because changes in nuclear spin alter long-lived entanglement within Ca_9_(PO_4_)_6_ clusters and thereby modulate downstream neural events. This scenario presupposes both the stable formation of Posner molecules and the preservation of multi-second ^31^P coherence (Fisher, 2015; Swift et al., 2018; Player and Hore, 2018). (ii) Radical-pair models instead explain magnetic and isotopic dependencies via spin-selective reaction yields in well-characterized radical pairs, without invoking Posner clusters. For example, entangled-radical mechanisms have been developed to account for lithium isotope effects and xenon anesthesia (Zadeh-Haghighi and Simon, 2021, 2022, 2023; Smith et al., 2021). (iii) More conventional routes, including differences in pharmacokinetics, protein binding, or receptor-level interactions, remain available for both lithium and xenon within standard anesthetic and psychopharmacological theory.^7^ Given this underdetermination, existing isotope data cannot be regarded as selective evidence for Posner-molecule entanglement; rather, they highlight the need for experiments specifically designed to discriminate among Posner-based, radical-pair, and classical mechanisms.

### 3.3 Axis II: philosophical sufficiency

Philosophically, the nuclear-spin hypothesis faces a “double-jump”: even if nuclear-spin entanglement influences neuronal activity, we still must explain how neural activity produces subjective experience. Thus Family B likewise leaves the hard problem unresolved, partitioning it into spin → neural effects, and neural effects → experience. The first step remains hypothetical; the second belongs to the traditional mind–brain problem and is not automatically solved by invoking quantum physics. For example, even if some nuclear-spin state corresponded to a neural pattern associated with focused attention, we would still need to explain why that neural pattern yields a particular qualitative feel, a challenge facing all physicalist theories. Proponents of the nuclear-spin route have largely deferred deeper philosophical analysis, tending to presume that once quantum entanglement in information processing is shown, the consciousness problem becomes “easier.” Strictly speaking, however, in the absence of a mapping from nuclear-spin quantum states to subjective states, philosophical sufficiency remains weak. In short, Family B offers a novel biophysical possibility, but has not yet integrated any theory of the nature of experience to close the explanatory gap. Even if successful, the nuclear-spin hypothesis would more likely serve as a supplementary mechanism in brain information processing rather than a direct account of why there is first-person experience.

### 3.4 Axis III: empirical testability

Among the quantum proposals considered here, the nuclear-spin hypothesis is notable for offering relatively concrete experimental handles, such as isotope substitution and manipulation of spin-related parameters, to test whether nuclear spins make a functional contribution to consciousness. As Fisher and colleagues suggest (Fisher, 2015; Ettenberg et al., 2020), a series of double-blind studies can be designed: (a) compare lithium isotopes with different nuclear spins on animal models (e.g., antidepressant or mania paradigms); (b) compare anesthetic potency across inert-gas isotopes (e.g., xenon) with different nuclear spins; and (c) use nuclear magnetic resonance (NMR) or electron paramagnetic resonance (EPR) to directly probe for abnormally long-lived Pi coherences in brain tissue. For example, low-temperature NMR on extracted brain samples could search for multi-quantum resonances at pre-specified frequencies to test for entangled states within Posner clusters. To strengthen the rigor of the research and transparency, such experiments should be pre-registered, specifying primary outcomes and statistical analyses in advance, and assessing sensitivity/power (discriminatory capacity). If spin effects exist but are not detected, one must distinguish insufficient sensitivity from theory failure; hence reports should include confidence intervals or upper bounds on effect sizes for null results (Zadeh-Haghighi and Simon, 2023). Multi-center collaboration is essential. For instance, parallel attempts to replicate lithium-isotope effects with open data for robustness checks. Publication of both positive and null findings should be encouraged to avoid file-drawer bias. Existing positive results (e.g., xenon and lithium isotope studies) warrant extension and verification, whereas existing negative results (e.g., calcium isotopes under anesthesia) should be retested across conditions to assess generality. In sum, while Family B remains controversial at its core, it offers rich testing avenues. The Family B program yields substrate-specific predictions targeting ^31^P nuclear-spin degrees of freedom. Systematic isotope experiments and spin-sensitive measurements could, in the coming years, decisively evaluate the program’s viability and provide firmer empirical footing for (or against) consciousness-related nuclear-spin effects.

## 4 Family C: macro-scale quantum-optical and MRI “non-classical signals”

Family C shifts attention to the macro scale, claiming observations of “non-classical” signals at the level of whole-brain imaging. This line was sparked by experiments from Kerskens and colleagues, who used specialized MRI(magnetic resonance imaging) sequences to detect heartbeat-locked zero-quantum coherence (ZQC) signals in the conscious human brain, interpreted as a macroscopic readout of nuclear-spin entanglement (Kerskens and López-Pérez, 2022). The report immediately triggered methodological debate: other researchers questioned whether these signals truly originate from quantum entanglement or instead from inadequately suppressed classical dipolar interactions or physiological artifacts (see Warren, 2023; Kerskens and López-Pérez, 2023). At present, the focus of the Family C program is more on establishing signal specificity and excluding artifacts than on directly advancing philosophical interpretation. If such macro-level non-classical signals are confirmed to exist, however, they would constitute strong evidence for quantum mechanisms participating in brain function and might offer a new observational window for consciousness research.

### 4.1 Thesis and predictions

Kerskens and López-Pérez (2022) report that, drawing on the idea of a “quantum witness” developed in quantum-gravity experiments (e.g., Bose et al., 2017; Marletto and Vedral, 2017), they designed an MRI sequence that suppresses conventional classical MR signals (e.g., single-quantum coherences). If signals remain detectable, they may be attributed to non-classical sources involving multiple-spin coherences, namely ZQC or related intermolecular multiple-quantum coherence (iMQC) signals. In scans of awake participants, they observed small oscillatory signals phase-locked to the cardiac cycle, with spectral characteristics consistent with ZQC (Kerskens and López-Pérez, 2022). The authors interpret this as a quantum signal generated by large numbers of proton-spin pairs in the brain, suggesting that the human brain may exploit quantum computation. Given the extraordinary nature of the claim, it has prompted methodological scrutiny and calls for independent replication (Warren, 2023; Kerskens and López-Pérez, 2023). To date, however, no independent peer-reviewed replication or confirmation of the reported heartbeat-locked ZQC signals has been published. A key methodological critique by Warren (2023) notes that MRI signals are highly susceptible to modulation by physiological cycles (heartbeat, respiration), making it crucial to distinguish putative quantum effects from ordinary physiological coupling. He argues that cardiac-induced brain pulsation, flow, and other classical mechanisms could generate ZQC-like signals, and that slight tweaks to sequence parameters can abolish or create such signals, indicating potential sequence-specific artifacts rather than robust evidence for macroscopic entanglement. In response, Kerskens and López-Pérez (2023) emphasize extensive controls for physiological noise and call for multi-center verification of the signal’s objective existence. The debate remains ongoing: the proposed non-classical brain signals are both exciting and contested, and more evidence is needed to decide the matter.

### 4.2 Axis I: physical feasibility

Detecting quantum signals with macro-scale brain MRI faces multiple physical challenges. First, MRI measures the macroscopic magnetization of hundreds of millions of nuclear spins; isolating entanglement effects within such signals is extremely difficult. The “witness” strategy used by Kerskens and López-Pérez (2022) suppresses typical dipolar and chemical-shift contributions via specialized pulse sequences, improving specificity. Yet to attribute the observed signals to a quantum source, all known classical mechanisms must be excluded, including gradient-field nonlinearities, unsuppressed flow effects, and magnetic-susceptibility artifacts, which are well-established sources of MRI artifacts (Graves and Mitchell, 2013; Krupa and Bekiesińska-Figatowska, 2015; Ho et al., 2023). Standard strategies in advanced MRI for mitigating such confounds include cardiac and respiratory gating, navigator echoes, careful gradient calibration, and phantom controls (Krupa and Bekiesińska-Figatowska, 2015; Ho et al., 2023). Kerskens and López-Pérez (2022) employ cardiac locking and sequence designs intended to suppress conventional coherences, but Warren (2023) argues that additional controls would be required to rule out long-range dipolar fields and intermolecular multiple-quantum coherences as classical sources (Richter and Warren, 2000; de Sousa et al., 2003). Whether the implemented protocol suffices to exclude these mechanisms remains an open technical question, with Warren (2023) maintaining that conventional mechanisms and sequence-dependent effects may account for the reported heartbeat-locked ZQC signals, and Kerskens and López-Pérez (2023) contending that their controls are adequate and calling for multi-center verification. Hence, the key to verifying physical specificity lies in control designs: for example, running the identical sequence on inanimate phantoms should not produce similar signals; altering sequence parameters (e.g., echo time, filtering window) should not fabricate artifacts or erase genuine signals; and testing in living organisms under unconscious states (deep anesthesia or post-mortem) should markedly reduce or abolish the signal if it is tied to consciousness-related quantum effects. Current discussion centers on whether the purported ZQC signals are genuine entanglement witnesses or can instead be explained by higher-order classical coherences generated by long-range dipolar fields and intermolecular multiple-quantum coherences, which are well characterized in the NMR/MRI literature (Richter and Warren, 2000; de Sousa et al., 2003; Chin et al., 2003; Warren, 2023). Kerskens and López-Pérez (2023) argue that their sequence minimizes such contributions, but this point remains under active debate. Finer-grained physical analyses and experiments are required. For instance, using higher field strengths or different nuclei to test dependence on quantum characteristics (such as selection rules for nontrivial spin superpositions). Overall, Family C has not yet earned broad acceptance on physical feasibility; stringent controls and replicable experiments are needed to establish that these macro signals truly instantiate quantum effects.

### 4.3 Axis II: philosophical sufficiency

Even if macro-level non-classical MRI signals are confirmed, their relation to consciousness still requires an independent argument. The Family C program currently emphasizes finding quantum phenomena, without explaining why such phenomena would yield subjective experience. One might speculate that, once a global entanglement signal in the brain is confirmed, new theoretical ideas could arise, or example, that consciousness is related to the dynamics of a unified or highly integrated brain-wide entangled spin state (Simon, 2019; Hu and Wu, 2004). Such conjectures, however, must rest on solid empirical grounding and rigorous philosophical reasoning. To date, the contribution of Family C is chiefly methodological: it offers a new potential tool for observing quantum effects relevant to consciousness. If future work shows that these signals indeed correspond to conscious states (e.g., present only during wakefulness, or varying with conscious content), the philosophical upshot would at least be that processes tied to consciousness may exceed classical computational models and require quantum descriptions. Still, in the absence of a “bridge principle,” we should remain cautious: quantum brain signals are not themselves an explanation of consciousness, at most evidence of physical correlation.^8^ Philosophical sufficiency awaits further theoretical work integrating such findings into a comprehensive account of consciousness.

### 4.4 Axis III: empirical testability

For Family C, the immediate priority is independent replication and multi-center validation. Given the controversy surrounding the initial series of reports by Kerskens and López Pérez (2022, 2023), multiple imaging centers should adopt the publicly released MRI sequence, apply it to diverse participant cohorts, and use a uniform analysis pipeline. This requires *a priori* criteria, e.g., spectral features of the signal, phase relationship to the cardiac cycle, (signal-to-noise ratio) SNR thresholds, to be registered before blind analysis. During experiments, all hardware and sequence details should be carefully logged, and raw data and code should be released for community scrutiny. The paradigm can be expanded to strengthen the evidential chain. The existing work already suggests that the heartbeat-locked signals are present during wakefulness but diminish with loss of consciousness, and that certain complexity measures correlate with cognitive performance, including working memory scores (Kerskens and López-Pérez, 2022; López Pérez et al., 2023). Building on these findings, future preregistered studies should systematically compare the same sequence across wakefulness vs. anesthesia or sleep and, within participants, manipulate task engagement or conscious content (e.g., report vs. no-report) to test whether such relationships are robust, specific, and resistant to classical physiological explanations. If the signals truly track levels or contents of consciousness and classical physiological parameters cannot account for the association, support for quantum approaches would be greatly strengthened. Conversely, repeated failures to detect similar signals would suggest that the original finding was a false positive. Additional macro-readouts should also be explored: optical methods to seek signatures of entanglement at the tissue level, or magnetic sensors to detect weak spin-correlated fields directly. Cross-validation among these techniques would yield a more comprehensive empirical assessment. Notably, the microtubule-optical measurements developed in Family A (e.g., fluorescence lifetime techniques) can be viewed as another macro-level readout with the same goal as Family C’s goal of capturing quantum effects at the system level. This suggests a future combined approach: simultaneously record brain magnetic signals, MRI signals, and microtubule-linked optical signals to examine whether they co-vary with consciousness. In short, empirical testability is decisive for Family C: only through rigorous multi-site, multi-modality verification can we determine whether reproducible quantum fingerprints truly exist at the macro scale of the human brain.

## 5 Discussion

### 5.1 Cross-family synthesis

From a comparative standpoint, the three Families show different strengths and weaknesses on the three evaluative axes. For physical feasibility, Family A has emerging evidence for collective electronic and vibrational effects in microtubules, but it still needs to show that these effects are robust and functionally relevant *in vivo*. Family B focuses on ^31^P nuclear spins in putative Posner clusters, which are theoretically appealing but experimentally uncertain in their existence, symmetry, and coherence lifetimes. Family C invokes macroscale, entanglement-like imaging signals without a specified microscopic substrate, so its physical interpretation is currently the most controversial. For philosophical sufficiency, all three Families still lack fully explicit psychophysical bridge principles. Recent work has strengthened Family A on this front, but Families B and C remain less fully developed at the level of psychophysical principles. For empirical testability, Family A and Family C already provide concrete readouts: microtubule-linked optical signals and anesthetic modulation in A, and heartbeat-locked MRI signatures in C. Family B offers some of the clearest decision procedures, via isotope substitution and spin-sensitive measurements, but relatively few such tests have been performed so far. Overall, no Family dominates on all three dimensions. Each instead offers a different entry point for future empirical and theoretical work.

Across these three approaches there are also shared limitations. The core philosophical challenge is still the same: none of the Families yet explains how candidate quantum processes give rise to the what-it-is-likeness aspect of experience, even when they offer more detailed psychophysical frameworks. Quantum mechanisms therefore look, at the current stage, more like potential realizers of consciousness than like complete theories of consciousness. On the physical side, both Families A and B must confront environmental decoherence in warm, wet tissue, and Family C must show that its macro-level signals survive increasingly strict artifact controls. On the methodological side, all three Families now have pathways for experimental progress, but they also depend on systematic replication, registered null results, and open data before any decisive verdict is possible. The synthesis, then, is mixed: quantum consciousness research has moved from almost pure speculation to concrete, testable programs, yet it still falls short of closing the explanatory gap and will require both stronger experiments and deeper philosophical work (Table 1).

**Table 1:** Cross-family schematic map across three evaluative axes.

Family	Physical feasibility	Philosophical sufficiency	Empirical testability
A	Emerging support (in vivo robustness to be determined)	Most developed (bridge still incomplete)	Concrete probes (anesthetic/optical readouts)
B	Uncertain substrate (coherence debated)	Promissory (bridge absent)	Clear tests (evidence limited)
C	Most controversial (micro-basis unclear)	Promissory (bridge absent)	Concrete paradigm (replication/artifacts)

### 5.2 Methodological recommendations

Given the contentious nature of quantum consciousness research, we emphasize stricter scientific norms to ensure quality and cumulativity. Equally important, we explicitly encourage independent laboratories (including critics) to reproduce the most contentious protocols and attempt decisive falsification rather than ignoring them. Pre-registration is essential: teams should publicly specify hypotheses, designs, and primary analyses before running experiments, preventing *post hoc* selection biases. Especially for high-noise measurements (e.g., weak-signal detection), predefining what outcomes count as support versus non-support can reduce subjective interpretation. Next, we recommend reporting a study’s discriminatory power (statistical power and effect sizes): when an expected effect is not detected, researchers should state below what effect size their design cannot discriminate, informing future study planning. Third, null results should be encouraged. Although journals often prefer positive findings, in an exploratory frontier the failure to replicate initial results is itself valuable. For example, if multiple groups repeat the MRI experiment reported by Kerskens and López Pérez (2022) and consistently find nothing, this matters for theory evaluation and should be publishable (e.g., as registered reports or data papers) to ease concerns about no-result studies. To lower risk and guarantee publishability irrespective of outcome, registered reports, adversarial collaborations, and multi-center consortia are recommended (Munafò et al., 2017; Chambers, 2013; Nosek and Lakens, 2014). Fourth, open-science practices are crucial: share raw data, analysis code, and materials so independent groups can test robustness or conduct meta-analyses. This is especially important in a cross-disciplinary field like quantum consciousness, where experts from different domains can help identify artifacts or propose refinements. With rigorous methodological standards, debates can shift from protracted authenticity quarrels to constructive hypothesis testing, accelerating the pruning of ineffective hypotheses and the accumulation of credible evidence.

### 5.3 Philosophical significance and a cautious outlook

The philosophical import of quantum consciousness research depends on whether we can locate a path to subjective experience. At the current stage, even if certain quantum mechanisms in the brain are verified, we should remain modest: at best, such findings offer new insights into realization rather than an ultimate account of the nature of consciousness. As some philosophers warn, the concept of “quantum” should not be treated as a panacea for the hard problem (Searle, 1997), invoking quantum mechanics does not automatically resolve the question of what-it-is-likeness. A genuine understanding of consciousness still requires synthesizing neuroscience, cognitive science, and philosophy. If quantum-level mechanisms exist, they may imply computational capacities beyond classical Turing models, perhaps even hinting that the relation between consciousness and the basic laws of physics is deeper than expected. Even with emerging proposals of psychophysical bridging laws—for example, Wiest and Puniani’s (2025) Orch OR based framework, which links unified quantum reductions to unified conscious moments—the standards for a fully explicit Chalmers-style set of laws that necessitate fine-grained phenomenal character from physical facts have not yet been met. It would therefore be premature to infer, at this stage, robust metaphysical conclusions (e.g., that consciousness is simply a manifestation of some cosmic quantum field) from preliminary quantum-mechanical findings alone. Instead, quantum discoveries should be viewed as supplementing the question of how the brain works, while the question of what consciousness is still requires independent inquiry. Should quantum consciousness research make substantive progress, philosophers will likely need to update theories of mind. For example, considering whether consciousness corresponds to a novel physical informational structure. Until then, our stance should be open yet testability-first, lest quantum views devolve into a new kind of mysticism. Our caution here is not a dismissal of such bridging proposals, but a call to treat them as promising but as-yet incomplete candidates that must be assessed alongside, and in dialogue with, independent philosophical theories of mind.

### 5.4 Limitations and future directions

This review has limitations that follow from its design. First, the selection is intentionally narrow. We focus on proposals that specify a concrete neural/biological substrate and a plausible route to empirical testing. We therefore exclude many broader “quantum mind” views that are mainly interpretive, metaphorical, or cosmological. Those approaches would require a different taxonomy and different evaluation criteria. Second, the three-axis framework is not exhaustive. It prioritizes mechanism and discriminability. It gives less space to other dimensions, such as computational constraints, formal model comparison, or theory choice under uncertainty. Future work could refine the framework or add complementary dimensions. Third, the evidential base is incomplete. Publication bias and selective visibility of null results remain concerns. Some relevant work may also be missed due to language and database coverage.

These limits suggest clear directions. Future reviews should update the evidence as replications and registered null results accumulate. Comparative work should also evaluate quantum and non-quantum theories under shared criteria. Finally, progress will benefit from interdisciplinary, co-designed studies that connect physics-level claims to neuroscience, psychology, and philosophical constraints.

## 6 Conclusion

In sum, under our three-fold standard of physical feasibility, philosophical sufficiency, and empirical testability, no quantum approach to consciousness presently satisfies all requirements convincingly. Nevertheless, each route offers valuable insights: microtubule-based models (Family A) highlight the potential importance of subcellular ultrastructure; the nuclear-spin hypothesis (Family B) draws attention to often-overlooked nuclear degrees of freedom; and reports of macro-scale quantum signals (Family C) invite reconsideration of phenomena that may have been neglected in large-scale representations of brain activity. Caution means acknowledging contradictions and limitations in existing theories and data without overstating preliminary results; progress means not ruling out reasonable quantum mechanisms prior to falsification, but instead designing more rigorous tests. Empirical testability should remain the primary criterion: only when a quantum claim is repeatedly supported across empirical tests can it be regarded as a more well-established part of consciousness research. Conversely, if a line of work long lacks confirmation or is falsified, we should let it go. The riddle of consciousness remains profoundly complex: quantum mechanics may be one piece of the puzzle, but a solution will likely require sustained multidisciplinary collaboration. In the explorations ahead, progress should be guided by the scientific method, advancing with a balance of curiosity and skepticism.

## Footnotes

1 Here “bridge principle” is used in Chalmers’ strong sense of a psychophysical principle: a lawlike principle connecting physical processes with phenomenal properties, such that the complete physical facts, together with the relevant psychophysical principles, would suffice to determine the corresponding facts about experience. Without such a principle, a theory may describe physical correlates or mechanisms of consciousness, but it does not yet explain why or how determinate phenomenal character arises.

2 This is in fact a rather large and heterogeneous family, for example: construing consciousness as “embodied quantum entanglement” that grounds the biological origin and physiological unity of mind (Torday, 2023); claiming that mind actualizes quantum potentia so that qualia arise with collapse (Kauffman and Roli, 2023); asserting that the very occurrence of quantum superposition already yields experience and that the structure of the superposition determines qualitative character (Neven et al., 2024); and the cosmopsychist proposal of a ubiquitous field of consciousness (UFC)/quantum electrodynamics (QED) zero-point field as a “repository” of phenomenal qualities (Keppler and Shani, 2020; Keppler, 2021, 2024), etc. These various proposals share a common problem: they do not make mechanism-level commitments on the three points above. Accordingly, in this article they are mentioned only as background and are not included in our three-axis evaluation oriented to mechanism-and-evidence commitments.

3 The Orch OR framework originally did not consider anesthesia to act solely on microtubules (Hameroff and Penrose, 2014; Craddock et al., 2015; Craddock et al., 2017) mainly because soluble anesthetics like ketamine, propofol and etomidate seemed to act on membrane receptors. However Meyer-Overton predicts all anesthetics act on the same target, and results now suggest ketamine, propofol and etomidate do bind and act on microtubules to cause anesthesia, and also bind and have effects on membrane receptors (Craddock et al., 2015; Craddock et al., 2017).

4 There is considerable empirical support for “flicker” in the specific sense of discrete, non-overlapping temporal windows of perceptual integration, particularly in vision and attention (VanRullen and Koch, 2003; VanRullen, 2016). Wiest and Puniani (2025) situate Orch OR within this framework, suggesting that *γ*-frequency Orch OR events could provide a mechanistic basis for such discrete perceptual cycles, thereby weakening the intuitive “flicker” objection. However, (a) these findings primarily concern the temporal organization of perceptual consciousness and do not by themselves extend to the full range of conscious phenomena; and (b) even if discrete windows are granted, they do not yield a derivation of the qualitative character of what it is like from microtubule-level or quantum-level dynamics without an additional psychophysical bridge principle. Thus, the evidence for discrete perceptual cycles is at most structurally compatible with, and mildly supportive of, Orch OR, but it does not by itself establish the philosophical sufficiency of the theory.

5 For example, lithium’s psychotropic effects are plausibly mediated by several well-established “classical” mechanisms, including inhibition of glycogen synthase kinase-3 (GSK-3), inhibition of inositol monophosphatase, and modulation of intracellular signaling cascades and neurotrophic pathways (Quiroz et al., 2010; Chiu and Chuang, 2010). These routes suffice to explain many lithium effects on neural function, so lithium isotope findings cannot be straightforwardly taken as positive evidence for Posner-based mechanisms.

6 ^3−^To avoid confusion: throughout Family B, “Pi” follows Fisher (2015) and denotes inorganic phosphate (PO₄); the relevant quantum degree of freedom is the phosphorus-31(^31^P) nuclear spin (I = 1/2) within putativeCa_9_(PO_4_)_6_ (“Posner”) clusters. This is distinct from the *π*-electronic (“π-resonance”) excitations in aromatic residues (e.g., tryptophan, tyrosine, phenylalanine) discussed under Family A in the context of microtubules and anesthetic binding (Craddock et al., 2014; Craddock et al., 2017). We retain “Pi” here to match Fisher’s notation, but it should not be conflated with π-electron phenomena.

7 for lithium, see mechanistic reviews on GSK-3/inositol pathways and broader signaling (Quiroz et al., 2010; Chiu and Chuang, 2010; Snitow et al., 2021); for xenon, see classical targets such as N-methyl-D-aspartate(NMDA)receptors and nicotinic acetylcholine(nACh)receptors, and related channel effects (Haseneder et al., 2009; Franks, 2008; Dickinson and Franks, 2010). Evidence for specific anesthetic protein-binding sites and target mapping via crystallography/photoaffinity labeling further supports such classical mechanisms (Vedula et al., 2009; Woll et al., 2018).

8 In the stronger Chalmers-style sense defined earlier, current Family C work does not itself supply a bridge principle.It offers putative signatures of non-classical dynamics without specifying laws that fix determinate conscious states. Recent work by Wiest and Puniani (2025) can be read as advancing a candidate set of psychophysical principles for an Orch OR–style microtubular substrate, linking certain unified Orch OR states to unified conscious moments and thereby strengthening the prospects of Family A, and macro-scale entanglement-like signals of the kind reported by Kerskens and López-Pérez (2022) could, if robustly confirmed and reliably tied to such states, be interpreted as empirical support consistent with that framework. However, Wiest’s proposal remains partly programmatic and does not yet yield a fully explicit set of psychophysical laws that necessitate fine-grained phenomenal character from physical facts alone; our cautionary claim here is directed at this stronger standard.
